# Comparative safety and short-term outcomes of intraoperative radiotherapy versus neoadjuvant chemoradiotherapy in locally advanced rectal cancer: a retrospective study

**DOI:** 10.3389/fsurg.2025.1713146

**Published:** 2026-01-02

**Authors:** Zhijie Gong, Yingze Li, Jun Zhou, Yanjie Deng, Yinghao He, WeiWei Wang, Qiangbang Yang, Jian Pan, Minghui Ma

**Affiliations:** 1 The First School of Clinical Medicine, Southern Medical University, Guangzhou, China; 2 Second Department of Gastrointestinal Surgery, Maoming People’s Hospital, Maoming, China; 3 The First Clinical College of Medicine, Guangdong Medical University, Zhanjiang, China

**Keywords:** locally advanced rectal cancer, intraoperative radiotherapy, neoadjuvant chemoradiotherapy, safety and efficacy, retrospective study

## Abstract

**Purpose:**

Neoadjuvant chemoradiotherapy (nCRT) constitutes an integral component of the standard therapeutic strategy for locally advanced rectal cancer (LARC) but carries cumulative toxicity, cost, and occasional non-response. Intraoperative radiotherapy (IORT) delivers a single high dosage directly to the tumor bed and may overcome these limitations. We compared short-term efficacy and safety of low-kilovoltage(kV) x-ray IORT with long-course nCRT.

**Methods:**

LARC patients treated at Maoming People's Hospital (2022–2024) were retrospectively reviewed. The nCRT cohort received 45–50 Gy radiotherapy plus capecitabine before surgery; the IORT cohort underwent INTRABEAM low-kV x-ray IORT (12.5–20 Gy) during surgery. Disease-free survival (DFS), overall survival (OS), postoperative complications, and recurrence were analyzed. Survival was estimated by Kaplan–Meier curves.

**Results:**

A total of 67 patients were included (46 in nCRT, 21 in IORT). Kaplan–Meier analysis showed no significant difference in DFS or OS between the IORT and nCRT groups (DFS *P* = 0.669; OS *P* = 0.864). 3-year DFS (53.7% vs. 52.8%, *P* = 0.669) and OS (89.0% vs. 78.4%, *P* = 0.864) did not differ between IORT and nCRT. Early postoperative bowel obstruction and urinary retention were more frequent after IORT, although neither difference was significant; long-term complications and recurrence patterns remained comparable between the groups.

**Conclusion:**

Single-fraction low-kV IORT provides short-term survival equivalent to standard nCRT with acceptable perioperative safety. It is a viable option for LARC patients unable or unwilling to undergo prolonged nCRT, although vigilance for early gastrointestinal and urinary complications is warranted.

## 1 Introduction

Colorectal cancer ranks third in global cancer incidence and is the second leading cause of cancer-related death worldwide (1). Approximately 70% of rectal cancer patients present with locally advanced rectal cancer (LARC) at initial diagnosis, defined by American Joint Committee on Cancer (AJCC) staging T3–4 and/or regional lymph node positivity without distant metastases (2, 3). The standard approach to treating LARC includes neoadjuvant chemoradiotherapy (nCRT) followed by total mesorectal excision (TME) (4). nCRT induces tumor regression, achieves downstaging, reduces the risk of recurrence, and ultimately improves patient survival (5).

However, approximately 20%–30% of LARC patients exhibit poor responsiveness to nCRT (6, 7). For these individuals, the prolonged nCRT regimen might permit tumor progression. Furthermore, a substantial fraction of LARC patients experience adverse effects during nCRT treatment, including leukopenia, diarrhea, and radiation dermatitis (8–10). Furthermore, radiotherapy-induced edema following nCRT can obscure anatomical landmarks, impede precise tissue dissection, and exacerbate peritumoral adhesions, thereby increasing operative complexity (11).

Intraoperative radiotherapy (IORT) administers a concentrated dose of radiation to the tumor site with precision during surgical procedures (12). Studies indicate that a single 20 Gy IORT dose achieves a cytotoxic effect comparable to conventional external beam radiation therapy of 40–60 Gy (13). Studies have demonstrated that IORT significantly reduces local recurrence risk among high-risk rectal cancer patients, particularly those with suspected positive margins (14). Consequently, several international guidelines have recommended IORT as a supplemental local boost for T4 tumors or cases with close/positive margins (15, 16).

Compared with nCRT, IORT offers some potential advantages: it eliminates the preoperative waiting period, thereby mitigating the risk of tumor progression and nCRT-related toxicities; reduces the economic burden; and avoids radiotherapy-induced bowel edema that can complicate surgery. However, the ability to predict response to nCRT before treatment remains limited, so in current clinical practice IORT cannot be used as a replacement strategy specifically targeting potential non-responders. In this context, as an exploratory, hypothesis-generating retrospective analysis, our study compares IORT combined with surgery vs. nCRT combined with surgery in LARC patients, evaluating short-term efficacy and safety outcomes in this specific real-world setting.

## 2 Materials and methods

### 2.1 Patients and design

**Figure 1:**
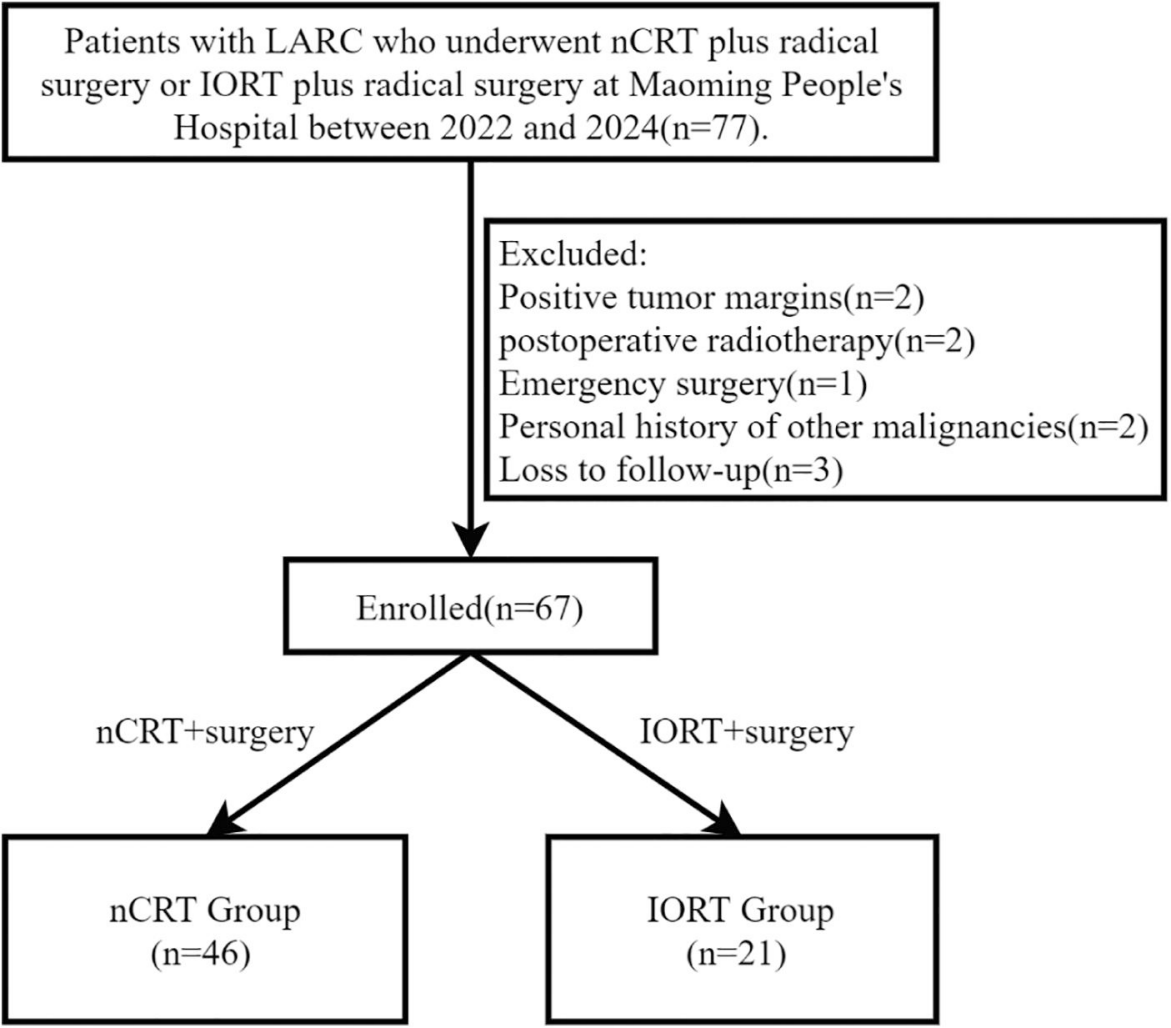
Flowchart of this research.

The overall study flowchart is presented in Figure 1. This retrospective study enrolled LARC patients who underwent either nCRT combined with surgery or IORT combined with surgery at Maoming People's Hospital from 2022 to 2024. All patients first underwent colonoscopy with biopsy to confirm the diagnosis of rectal adenocarcinoma. Local staging was based on high-resolution rectal MRI, together with contrast-enhanced chest and abdominal CT to exclude distant metastases. MRI scans were interpreted by experienced radiologists to assess cT and cN stage, mesorectal fascia status(MRF), extramural vascular invasion (EMVI), and involvement of the anal sphincter complex. In the nCRT group, repeat MRI was performed approximately 6–8 weeks after completion of neoadjuvant chemoradiotherapy to restage the tumor and evaluate the radiologic tumor regression grade. Inclusion criteria were: (1) age 18–75 years; (2) histopathologically confirmed rectal cancer; (3) no distant metastasis detected by systemic evaluation(M0); (4) Tumor staged by rectal MRI as cT3–4Nany or cT1–2N1–2, with the distal tumor margin located within 10 cm of the anal verge; (5) No contraindications to chemoradiotherapy; (6) ECOG performance status ≤1. Exclusion criteria included: (1) positive tumor margins; (2) postoperative adjuvant radiotherapy; (3) emergency surgery; (4) history of other malignancies; (5) loss to follow-up. Ethical approval was obtained from the Ethics Committee of Maoming People's Hospital (Approval No. PJ2025MI-K076-01).

### 2.2 Treatment

Patients with clinically diagnosed LARC were recommended to undergo nCRT, consisting of intensity-modulated radiotherapy delivering 45–50 Gy in 25–28 fractions (1.8–2.0 Gy per fraction) with concurrent capecitabine for 5–6 weeks, followed by surgery at 6–8 weeks post-treatment. All surgical procedures, including low anterior resection (LAR), intersphincteric resection (ISR), and abdominoperineal resection (APR), were performed by a single experienced surgical team in strict accordance with TME principles (17). For patients who accepted this standard treatment pathway, IORT was not offered as a treatment option at any stage.

The decision to create a prophylactic diverting ileostomy was made intraoperatively based on the surgeon's assessment of anastomotic risk, mainly considering low tumor location (5 cm or less from the anal verge), prior nCRT, bowel wall edema and tissue friability, mesenteric tension, and relevant patient comorbidities.

**Figure 2:**
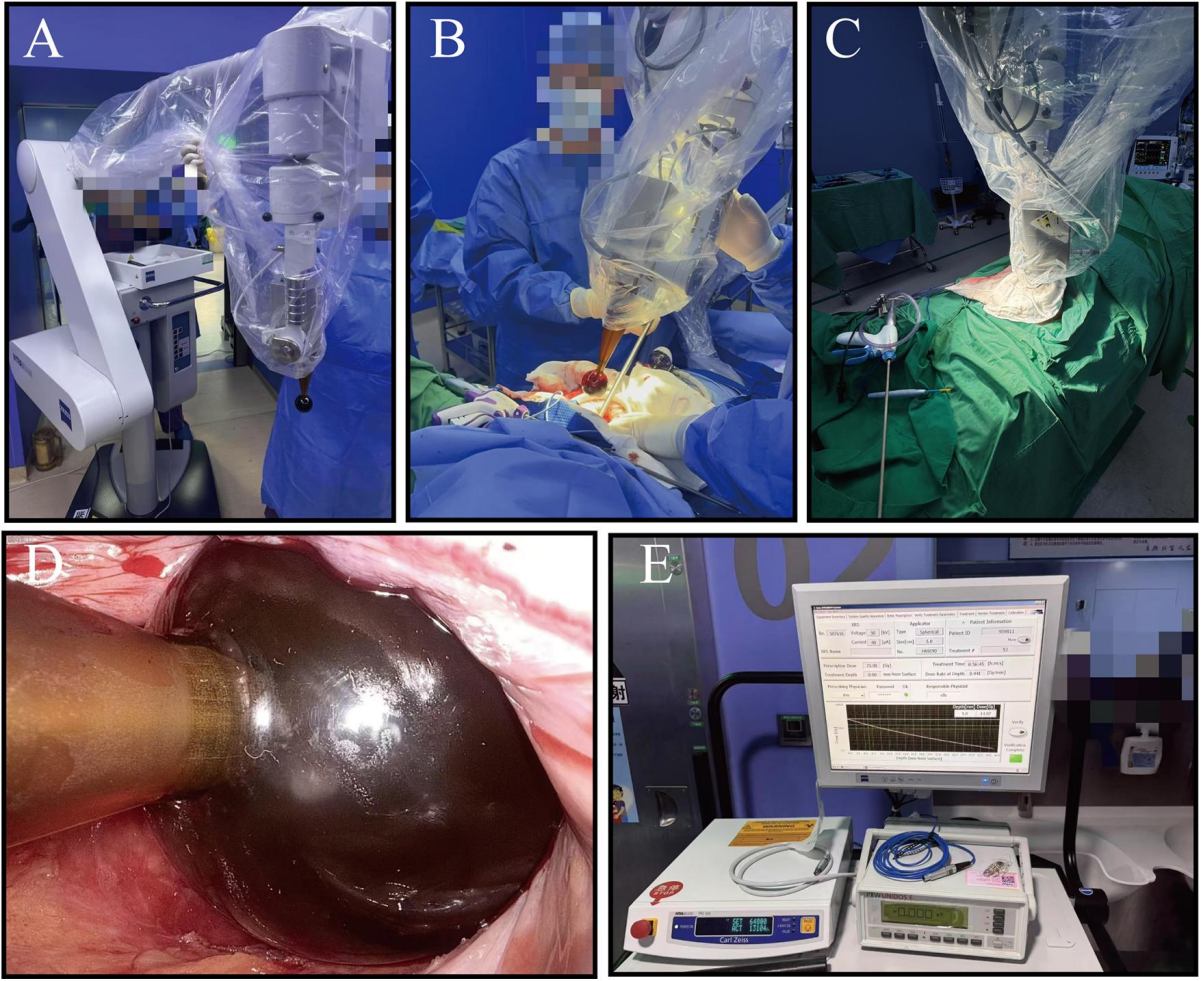
**(A)** The INTRABEAM main radiation unit; **(B,C)** Pre-radiotherapy preparation procedures; **(D)** precise positioning of the spherical applicator within the surgical cavity after tumor excision; **(E)** the INTRABEAM system control console.

IORT was considered only for patients who explicitly refused nCRT after full counselling. In these cases, patients were informed that IORT in this indication is not guideline-endorsed and that evidence regarding its efficacy and risks is still limited, and they could then choose either surgery alone or surgery combined with low-kV IORT. Intraoperative radiotherapy was delivered immediately after tumor resection using the INTRABEAM mobile radiotherapy system (Carl Zeiss Meditec AG, Jena, Germany), as illustrated in Figure 2. To conform to pelvic anatomy, circular applicators measuring 3.5–5 cm in diameter were typically selected. The radiation physicist and radiation oncologist determined the appropriate dose and irradiation parameters based on the size and shape of the exposed tumor bed or areas at risk for residual disease. Routine prescription doses ranged from 12.5 Gy to 15 Gy, whereas doses of 15 Gy–20 Gy were used for cases with close surgical margins or suspected residual malignancy. The reference depth for dose prescription was set between 0 and 3 mm, and moist gauze was applied to protect adjacent normal tissues. The irradiation procedure lasted 30–40 min. After completion of radiotherapy, intestinal anastomosis or prophylactic stoma formation was performed. Postoperative complications were recorded and their severity graded according to the Clavien-Dindo classification system.

Adjuvant chemotherapy regimens were determined according to postoperative pathological findings. Treatment typically commenced 1–2 months after surgery, and adjuvant therapy is recommended for all LARC patients. Patients with stage II disease or lower, or those intolerant of combination chemotherapy, received capecitabine monotherapy, while all other patients were treated with combination regimens of either CapeOX or mFOLFOX6.

### 2.3 Follow-up

Patients were followed every 3–6 months for the first 3 years, monitoring serum CEA and CA199 levels, and annually undergoing enhanced pelvic MRI and thoracoabdominal CT for 3 years. The primary endpoints were disease-free survival (DFS) and overall survival (OS), with follow-up concluding in May 2025.

### 2.4 Statistical analysis

Continuous variables were summarized as mean ± SD or median (interquartile range, IQR) and compared using *t*-tests or Wilcoxon tests. Categorical data were summarized as counts with corresponding percentages, and group differences were assessed using the *χ*^2^ test or Fisher's exact test, as appropriate. Survival curves were estimated using the Kaplan–Meier method with log-rank tests for group comparisons. For pairwise comparisons among multiple survival curves, *P* values were adjusted by the Holm method. Statistical significance was defined as *P* < 0.05, and analyses were conducted using R version 4.3.3 (https://www.r-project.org/).

## 3 Results

### 3.1 Patient characteristics

Table 1 presents the baseline clinical characteristics of patients grouped by treatment modality: nCRT (*n* = 46) and IORT (*n* = 21). Between the two groups, no significant differences were observed in gender (*P* = 0.502), age (*P* = 0.863), body mass index (BMI, *P* = 0.871) or ECOG performance status (*P* = 0.698). The prevalence of comorbidities (diabetes and hypertension) and tumor markers (CEA and CA19-9) was comparable (all *P* > 0.05). Likewise, MRI-defined clinical T stage (*P* = 0.637), N stage (*P* = 0.861), mesorectal fascia involvement (MRF, *P* = 0.729), extramural vascular invasion (EMVI, *P* = 0.299) and tumor diameter (*P* = 0.317) did not differ significantly between groups. However, tumors in the nCRT group tended to be located closer to the anal verge than those in the IORT group, although this difference did not achieve statistical significance (*P* = 0.085).

**Table 1:** Baseline clinical characteristics of enrolled patients.

Variables	nCRT	IORT	*P*
	(*n* = 46)	(*n* = 21)	
Gender			0.502
Female	17 (37%)	6 (28.6%)	
Male	29 (63%)	15 (71.4%)	
Age, years			0.863
Mea*n* ± SD	63.6 ± 9.4	63.9 ± 5.5	
BMI			0.871
Mean ± SD	23.2 ± 3.7	23 ± 3.6	
ECOG score			0.698
0	41 (89.1%)	18 (85.7%)	
1	5 (10.9%)	3 (14.3%)	
Comorbidity			
Diabetes	4 (8.7%)	2 (9.5%)	1
Hypertension	8 (17.4%)	5 (23.8%)	0.526
CEA			0.319
Negative	34 (73.9%)	13 (61.9%)	
Positive	12 (26.1%)	8 (38.1%)	
CA199			1
Negative	40 (87%)	18 (85.7%)	
Positive	6 (13%)	3 (14.3%)	
Clinical T stage^a^			0.637
T2	5 (10.9%)	4 (19%)	
T3	30 (65.2%)	13 (61.9%)	
T4	11 (23.9%)	4 (19%)	
Clinical N stage^a^			0.861
N0	16 (34.8%)	6 (28.6%)	
N1	13 (28.3%)	7 (33.3%)	
N2	17 (37%)	8 (38.1%)	
MRF^a^			0.729
Negative	22 (47.8%)	11 (52.4%)	
Positive	24 (52.2%)	10 (47.6%)	
EMVI^a^			0.299
Negative	20 (43.5%)	12 (57.1%)	
Positive	26 (56.5%)	9 (42.9%)	
Tumour diameter,^a^ cm			0.317
≤5	30 (65.2%)	11 (52.4%)	
>5	16 (34.8%)	10 (47.6%)	
Distance from anal verge,^a^ cm			0.085
≤5	30 (65.2%)	9 (42.9%)	
>5	16 (34.8%)	12 (57.1%)	

BMI, body mass index; CEA, carcinoembryonic antigen; CA-199, carbohydrate antigen 199; MRF, mesorectal fascia invasion; EMVI, extramural venous invasion; SD, standard deviation.

a MRI defined.

### 3.2 Treatment and pathological results

Table 2 compares treatment and pathological characteristics between the nCRT and IORT groups. Low anterior resection was the most common procedure, with over 90% of cases in both cohorts performed laparoscopically, and more than 75% of patients in each group receiving postoperative chemotherapy. Notably, the rate of prophylactic ileostomy was significantly higher in the nCRT group compared to the IORT group (89.1% vs. 52.4%, *P* = 0.002). This difference is likely driven mainly by the lower tumor location in the nCRT group. In this cohort, more tumors were located 5 cm or less from the anal verge, increasing anastomotic risk and making surgeons more inclined to create a protective stoma. nCRT-related bowel edema and tissue fragility may further reinforce this preference. Due to the effects of chemoradiotherapy on tumors, substantial differences were observed in pathological evaluations, including lower T-stage, N-stage, and reduced vascular and nerve invasion in the nCRT group, demonstrating the significant inhibitory impact of preoperative treatment on tumor infiltration. The pathological complete response (pCR) rate in the nCRT group was approximately 20%, with about 55% of patients achieving favorable tumor regression (TRG 0–1).

**Table 2:** Treatment and pathological characteristics of patients.

Variables	nCRT	IORT	*P*
	(*n* = 46)	(*n* = 21)	
Type of surgery			0.62
LAR	37 (80.4%)	16 (76.2%)	
APR	4 (8.7%)	1 (4.8%)	
ISR	5 (10.9%)	4 (19%)	
Surgical approach			0.584
Laparoscopic	44 (95.7%)	19 (90.5%)	
Open	2 (4.3%)	2 (9.5%)	
Preventive diverting ileostomy			0.002
Yes	41 (89.1%)	11 (52.4%)	
No	5 (10.9%)	10 (47.6%)	
Adjuvant chemotherapy			0.95
No	10 (21.7%)	5 (23.8%)	
Capecitabine	12 (26.1%)	4 (19%)	
CAPOX	16 (34.8%)	8 (38.1%)	
mFOLFOX6	8 (17.4%)	4 (19%)	
Pathologic T stage			0.039
T0	10 (21.7%)	0 (0%)	
T1	4 (8.7%)	0 (0%)	
T2	11 (23.9%)	7 (33.3%)	
T3	20 (43.5%)	14 (66.7%)	
T4	1 (2.2%)	0 (0%)	
Pathologic N stage			0.061
N0	33 (71.7%)	11 (52.4%)	
N1	11 (23.9%)	5 (23.8%)	
N2	2 (4.3%)	5 (23.8%)	
Histology			1
Non-Mucinous	43 (93.5%)	20 (95.2%)	
Mucinous	3 (6.5%)	1 (4.8%)	
Vascular invasion			0.17
No	34 (73.9%)	12 (57.1%)	
Yes	12 (26.1%)	9 (42.9%)	
Perineural invasion			<0.001
No	38 (82.6%)	9 (42.9%)	
Yes	8 (17.4%)	12 (57.1%)	
Pathologic complete response			—
Yes	9 (19.6%)	—	
No	37 (80.4%)	—	
Tumor regression grade			—
TRG 0	10 (21.7%)	—	
TRG 1	15 (32.6%)	—	
TRG 2	17 (37%)	—	
TRG 3	4 (8.7%)	—	

LAR, low anterior resection; APR, abdominoperineal resection; ISR, intersphincteric resection; CAPOX, capecitabine + oxaliplatin; FOLFOX, 5-FU + leucovorin + oxaliplatin.

### 3.3 Postoperative complications

Table 3 outlines postoperative complication differences between groups. Although perioperative safety in the IORT cohort remained acceptable overall, certain complications exhibited an increasing trend, underscoring the need for more rigorous perioperative risk-management strategies. Specifically, bowel obstruction (19.0% vs. 4.3%, *P* = 0.072) and urinary retention (14.3% vs. 2.1%, *P* = 0.087) were more frequent in the IORT group, suggesting potential adverse effects of IORT on bowel and bladder function. The mean operative time was longer in the IORT group than in the nCRT group (4.63 ± 1.12 vs. 3.16 ± 0.74 h, *P* < 0.001), reflecting the additional intraoperative radiotherapy procedure. Moreover, early postoperative complications of Grade II were more prevalent in the IORT group compared to the nCRT group (28.6% vs. 8.6%, *P* = 0.060), though the difference did not reach statistical significance. No perioperative mortality occurred in either group. However, each group had one Grade IV anastomotic leak with septicemia requiring ICU admission and reoperation. Overall, the incidence of anastomotic leakage in both groups remained within a normal, controllable range. Median postoperative hospitalization was approximately two days longer in the IORT group due to the increased trend in early complications. No significant differences were found in long-term complications such as anastomotic stenosis, parastomal hernia, and bowel or urinary/sexual dysfunction between groups (all *P* > 0.05), suggesting that intraoperative radiation primarily increases short-term postoperative risks.

**Table 3:** Postoperative complications in nCRT and IORT groups.

Variables	nCRT	IORT	*P*
	(*n* = 46)	(*n* = 21)	
Early complications (≤30 days)			
Anastomotic leaks	1 (2.1%)	1 (4.8%)	0.532
Anastomotic Bleeding	0 (0%)	1 (4.8%)	0.313
Bowel obstruction	2 (4.3%)	4 (19.0%)	0.072
Urinary Retention	1 (2.1%)	3 (14.3%)	0.087
Abdominal/pelvic infection	2 (4.3%)	1 (4.8%)	1
Wound infection	1 (2.1%)	1 (4.8%)	0.532
Lung infection	2 (4.3%)	0 (0%)	1
Urinary infection	1 (2.1%)	0 (0%)	1
Grading of complications (Claviene-Dindo)			
Grade II	4 (8.6%)	6 (28.6%)	0.060
Grade III	3 (6.5%)	2 (9.5%)	0.645
Grade IV	1 (2.1%)	1 (4.8%)	0.532
Overall	8 (17.4%)	9 (42.9%)	0.055
Postoperative Length of Stay			0.071
Median (IQR)	10 (9–12)	12 (10–16)	
Surgery duration			0.000
Mean (SD)	3.16 ± 0.74	4.63 ± 1.12	
Late complications (>30 days)			
Anastomotic Stricture	2 (4.3%)	2 (9.5%)	0.584
Anastomotic ulceration	2 (4.3%)	0 (0%)	1
Parastomal Hernia	1 (2.2%)	0 (0%)	1
Bowel obstruction	3 (6.5%)	0 (0%)	0.546
Defecatory Dysfunction	3 (6.5%)	3 (14.3%)	0.368
Urinary Dysfunction	2 (4.3%)	1 (4.8%)	1
Sexual Dysfunction	1(2.2%)	0(0%)	1

IQR, interquartile range; SD, standard deviation.

### 3.4 Survival analysis

During a median follow-up of 25 months (range, 10–37 months), disease recurrence or metastasis occurred in 11 patients (23.9%) in the nCRT group and 4 patients (19.0%) in the IORT group (Table 4). Specifically, the nCRT cohort exhibited two locoregional recurrences, five pulmonary metastases, three peritoneal metastases, and one bone metastasis, whereas the IORT cohort experienced one locoregional recurrence, one pulmonary metastasis, and two liver metastases—demonstrating comparable recurrence patterns between groups. Mortality during follow-up included one patient in the IORT group with liver metastasis and four in the nCRT group, of whom three had peritoneal metastases and one had pulmonary metastasis.

**Table 4:** Recurrence and metastasis rates in the nCRT and IORT groups.

Variables	nCRT	IORT	*P*
	(*n* = 46)	(*n* = 21)	
Local recurrence	2 (4.3%)	1 (4.7%)	1
Pulmonary metastases	5 (10.9%)	1 (4.7%)	0.657
Peritoneal metastases	3 (6.5%)	0 (0%)	0.546
Liver metastases	0 (0%)	2 (9.5%)	0.095
Bone metastases	1 (2.2%)	0 (0%)	1
Overall	11 (23.9%)	4 (19.0%)	0.76

**Figure 3:**
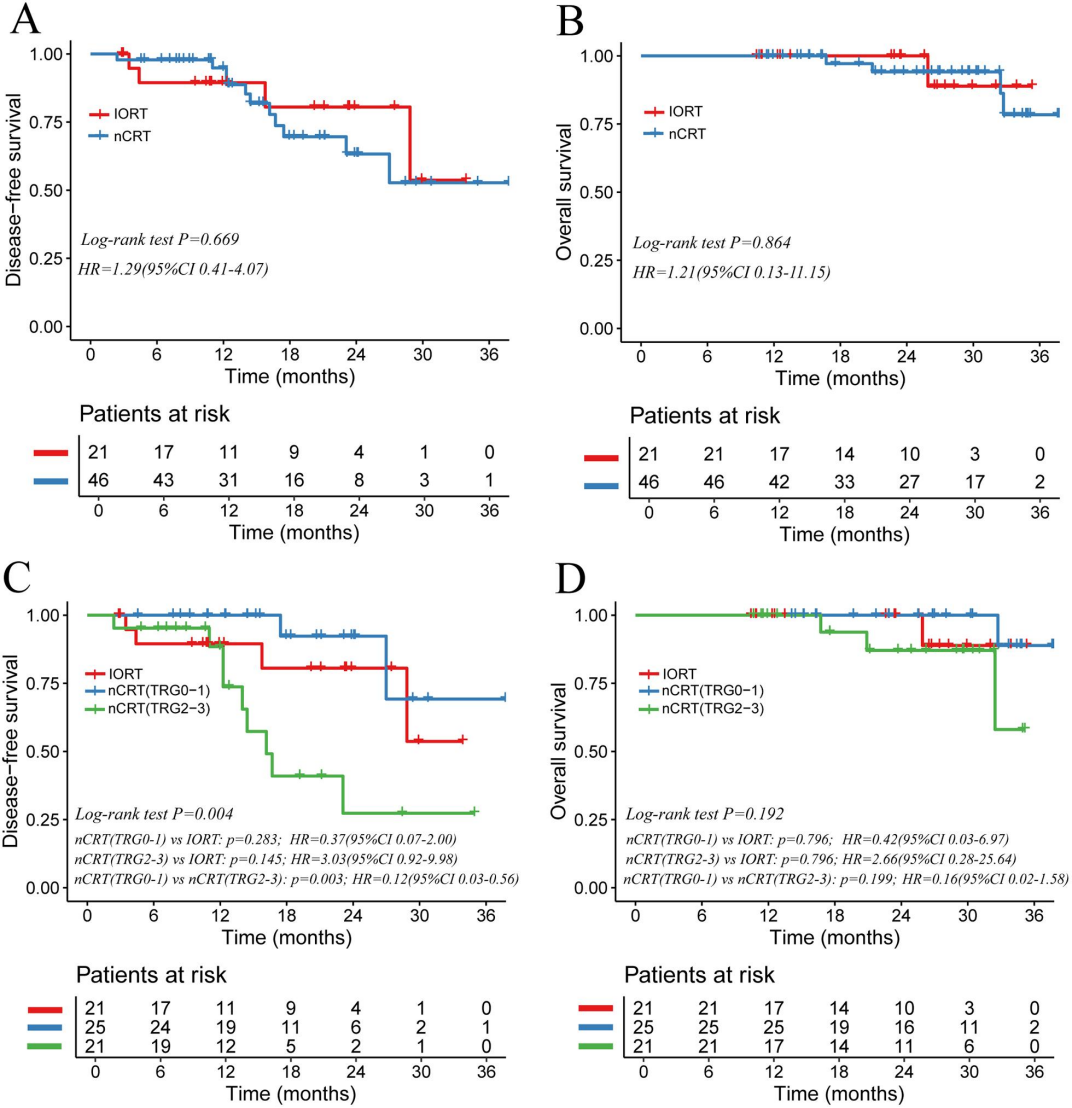
Kaplan–Meier curves comparing IORT and nCRT prognostic outcomes in locally advanced rectal cancer. **(A)** Disease-free survival (DFS) and **(B)** overall survival (OS) curves for IORT vs. nCRT; **(C,D)** DFS and OS curves further stratified by pathological tumor regression grade (TRG).

Kaplan–Meier survival curves (Figures 3A,B) showed no significant differences between nCRT and IORT groups in DFS (*P* = 0.669, HR = 1.29, 95% CI 0.41–4.07) or OS (*P* = 0.864, HR = 1.21, 95% CI 0.13–11.15). The 1-, 2-, and 3-year DFS rates were 89.5%, 80.5%, and 53.7% in the IORT group and 94.9%, 63.3%, and 52.8% in the nCRT group, respectively. Corresponding OS rates were 100%, 100%, and 89.0% for IORT and 100%, 94.1%, and 78.4% for nCRT, suggesting similar overall survival outcomes between the two strategies.

We further compared survival across IORT and nCRT subgroups stratified by TRG grade (Figures 3C,D). DFS differed significantly overall (*P* = 0.004). After Holm adjustment, the nCRT(TRG0-1) subgroup demonstrated a significant benefit over the nCRT(TRG2–3) subgroup (P_adj = 0.003; HR 0.12, 95% CI 0.03–0.56), whereas no significant differences emerged between the IORT cohort and either nCRT subgroup (P_adj > 0.05). The 1-, 2-, and 3-year DFS rates were 100%, 92.3%, and 69.2% in the nCRT(TRG0-1) subgroup, compared with 88.4%, 27.3%, and 27.3% in the nCRT(TRG2-3) subgroup, underscoring the positive impact of favorable pathological response on DFS. OS did not differ significantly among the three groups (log-rank *P* = 0.192), and Holm-adjusted pairwise comparisons likewise showed no statistical differences (P_adj > 0.05).

## 4 Discussion

Radical surgical resection remains the cornerstone treatment for LARC (15). Since Heald introduced TME in 1982, local recurrence rates have fallen from historical figures of 20%–40% to approximately 5%–10% (17, 18). Chemoradiotherapy has subsequently become an integral part of LARC treatment, enhancing tumor downstaging, R0 resection rates, and local control, thus reducing recurrence risks (19–21).

However, nCRT is not universally suitable. Some patients may poorly tolerate treatment toxicity, and some individuals decline therapy due to concerns about adverse effects or financial burdens. In such cases, IORT offers a viable alternative. Potemin et al. conducted an IORT study in a setting of limited radiotherapy equipment availability and demonstrated that IORT achieved satisfactory local control even in the absence of conventional preoperative radiotherapy (22). Therefore, IORT can serve as an immediate adjuvant radiotherapy, particularly valuable in resource-constrained or specific clinical scenarios.

Common IORT methods include intraoperative electron radiotherapy (IOERT), high-dose-rate IORT (HDR-IORT), and low-kilovoltage (kV) x-ray IORT using the INTRABEAM photon radiosurgical system. Low x-ray energy requires only minimal shielding, and the INTRABEAM system's compact, mobile design allows treatment directly in the operating room without patient transfer, greatly simplifying IORT implementation (23).

The efficacy and safety of IORT remain to be confirmed by large-scale randomized controlled trials. Most existing research has focused on IOERT or HDR-IORT, demonstrating improved local recurrence control. Liu et al. reported in a meta-analysis that IORT did not improve 5-year OS or DFS, but significantly enhanced local control (OR = 3.07, *P* < 0.001), supporting its role in reducing local recurrence (24). Mirnezami et al. similarly reported that IORT significantly reduced local recurrence risks to about one-third (25). Studies on low-kilovoltage x-ray IORT remain relatively limited. Guo et al. retrospectively analyzed 42 patients with locally advanced or recurrent rectal cancer, noting a 3-year OS of 49% and pelvic recurrence rate of 32% (26). These outcomes differ from our findings (88.9% 3-year OS and 4.7% local recurrence), likely due to their higher-risk patient population (45% with positive margins). Li et al. reported long-term results from 69 patients treated with low-kV IORT, noting 3-year OS of 89.4%, DFS of 71.5%, local recurrence of about 26%, and distant metastasis of 23% after nearly four years of follow-up (27). That study's three-year OS mirrored ours at about 89%, yet its DFS and local control edged ahead—likely a consequence of its longer follow-up and allowance for postoperative radiotherapy.

International guidelines recognize IORT as a method for enhancing local control in high-risk LARC cases (15, 16), and our study offers single-center evidence from an Asian cohort supporting this strategy's comparable efficacy to nCRT. Furthermore, we compared prognostic outcomes between the IORT cohort and the nCRT subgroups stratified by TRG grade. The IORT group's survival curves fell between the TRG 0–1 and TRG 2–3 groups, suggesting IORT might offer superior outcomes compared to prolonged nCRT for poorly responding patients. In recent years, multi-omics integration coupled with machine learning has demonstrated encouraging accuracy in predicting post-nCRT pCR status and TRG grades, paving the way for clinical decision-support tools (28–30), As these predictive models continue to improve, we propose that early radical surgical resection with IORT may outperform conventional nCRT in patients anticipated to have non-pCR or high TRG grades. It should be specifically emphasized that, nCRT remains the standard of care for LARC and plays a key role in achieving tumour downstaging and enabling sphincter-preserving approaches in many patients. Our intention is not to propose IORT as an equivalent alternative to standard nCRT. Rather, we aim to present a real-world observation that, in a subset of LARC patients who declined guideline-recommended nCRT for various reasons but received surgery with IORT, early survival at the current follow-up did not appear clearly inferior to that of patients treated with nCRT. These findings are exploratory and hypothesis-generating only and should not be interpreted as evidence to replace nCRT with IORT in routine practice.

The safety of IORT is another key concern. Previous studies reported overall postoperative complication rates following IORT ranging from approximately 15% to 59%, with common complications including wound-related issues, bowel dysfunction, urinary retention, and neuropathy (25). Li et al.'s retrospective analysis reported zero mortality within 30 days post-operation, with complications including anastomotic leakage at 7.25%, and urinary retention and intestinal obstruction each occurring in about 6% of cases (27). Another study examining low-energy x-ray IORT in pT3 colon cancer reported zero perioperative mortality within 30 days and a complication rate of 9% (31). In our cohort, 9 of 21 patients in the IORT group (42.9%) experienced Clavien–Dindo grade II or higher perioperative complications, which appears high as a proportion but reflects a small absolute number of events. Among these 9 patients, 6 had grade II complications, mainly transient bowel obstruction or urinary retention that were controlled with medication or simple interventions, and only 3 had grade III or higher complications. These findings suggest that the slightly higher overall complication rate in the IORT group is mainly driven by manageable low grade events rather than by a clear increase in severe, disabling or fatal complications. No significant differences were observed in long-term complications between groups, suggesting that IORT-related risks mainly involve increased mild early postoperative complications.

It should also be noted that toxicities related to nCRT, such as leukopenia, diarrhea and radiation dermatitis, are common in clinical practice. From the perspective of the entire treatment course, patients in the nCRT group often experience grade I–II toxicities before surgery, which are usually considered acceptable when adequate tumour downstaging and local control are achieved. Similarly, if the efficacy of IORT is confirmed in future studies, a modest increase in low grade postoperative complications may also be considered acceptable in clinical practice. Taken together, INTRABEAM low-energy x-ray IORT in this setting showed a manageable short-term safety profile in our cohort, with most additional events being low grade and controllable, although clinicians should remain vigilant regarding potential risks of intestinal obstruction and urinary retention, and these observations need to be confirmed in larger prospective cohorts.

This study has several limitations. Firstly, the IORT cohort consisted exclusively of patients who declined guideline-recommended nCRT, so selection bias is inevitable. Secondly, the relatively small sample size and limited follow-up duration reduce statistical power and may mask clinically relevant differences in long-term outcomes. Future larger-scale, multicenter prospective studies are required to validate our findings and enhance result robustness.

## 5 Conclusion

This study compared the efficacy and safety of INTRABEAM low-kV x-ray IORT with nCRT in LARC treatment. Our findings indicate that IORT achieves short-term DFS and OS comparable to nCRT, with comparable perioperative complication rates. However, clinicians should monitor potential increases in bowel obstruction and urinary retention risks. Low-kV x-ray IORT appears to be an effective adjunctive local therapy strategy for rectal cancer.

## Data Availability

The raw data supporting the conclusions of this article will be made available by the authors, without undue reservation.
